# The Kardashian index: a measure of discrepant social media profile for scientists

**DOI:** 10.1186/s13059-014-0424-0

**Published:** 2014-07-30

**Authors:** Neil Hall

**Affiliations:** Centre for Genomic Research, University of Liverpool, Liverpool, L69 7ZB UK

## Abstract

In the era of social media there are now many different ways that a scientist can build their public profile; the publication of high-quality scientific papers being just one. While social media is a valuable tool for outreach and the sharing of ideas, there is a danger that this form of communication is gaining too high a value and that we are losing sight of key metrics of scientific value, such as citation indices. To help quantify this, I propose the ‘Kardashian Index’, a measure of discrepancy between a scientist’s social media profile and publication record based on the direct comparison of numbers of citations and Twitter followers.

## Introduction

There are many scientists who, with hindsight, did not get much recognition for their achievements while they were alive. Consider Mary Anning, a fossil collector and paleontologist who lived in the early 19th century. Her meticulous recording and prolific findings contributed to the fundamental changes in our understanding of natural history, including the accepted view of extinction events. Yet, because of her sex and religious beliefs, much of her work was never recognized by her peers, and I expect you have never heard of her. Or Ada Lovelace, the daughter of Lord Byron, who is credited with writing the first ever computer program for the Analytical Engine, a mechanical computer designed by Charles Babbage. Despite her contribution, and obvious genius, she is much less well known than her male contemporaries. For a long time, the same could be said of Rosalind Franklin, whose work on determining the structure of DNA was largely ignored until years after her death.

It may be no coincidence that all of these overlooked heroes were women. I will return to this later.

Now consider Kim Kardashian; she comes from a privileged background and, despite having not achieved anything consequential in science, politics or the arts (although apparently she does have a scientific mind [1]), she is one of the most followed people on twitter and among the most searched-for person on Google. Her notoriety is said to have stemmed from an inadvertent internet release of a video featuring her and a boyfriend in a private moment. While her Wikipedia entry describes her as a successful businesswoman [2], this is due most likely to her fame generating considerable income through brand endorsements. So you could say that her celebrity buys success, which buys greater celebrity. Her fame has meant that comments by Kardashian on issues such as Syria have been widely reported in the press [3]. Sadly, her interjection on the crisis has not yet led to a let-up in the violence.

I am concerned that phenomena similar to that of Kim Kardashian may also exist in the scientific community. I think it is possible that there are individuals who are famous for being famous (or, to put it in science jargon, renowned for being renowned). We are all aware that certain people are seemingly invited as keynote speakers, not because of their contributions to the published literature but because of who they are. In the age of social media there are people who have high-profile scientific blogs or twitter feeds but have not actually published many peer-reviewed papers of significance; in essence, scientists who are seen as leaders in their field simply because of their notoriety. I was recently involved in a discussion where it was suggested that someone should be invited to speak at a meeting ‘because they will tweet about it and more people will come’. If that is not the research community equivalent of buying a Kardashian endorsement I don’t know what is.

I don’t blame Kim Kardashian or her science equivalents for exploiting their fame, who wouldn’t? However, I think it’s time that we develop a metric that will clearly indicate if a scientist has an overblown public profile so that we can adjust our expectations of them accordingly. In order to quantify the problem and to devise a solution, I have compared the numbers of followers that research scientists have on twitter with the number of citations they have for their peer-reviewed work. This analysis has identified clear outliers, or Kardashians, within the scientific community. I propose a new metric, which I call the ‘Kardashian Index’, which allows a simple quantification of the over, or under, performance of a scientist on social media.

## Methods

In this preliminary proof-of-concept study, I selected research scientists and recorded their number of followers. I did not devise a clever way of doing this randomly (after all this is just a bit of fun) but tried to pick a randomish selection of 40 scientists. I used Web of Knowledge to get citation metrics on these individuals. Obviously, there are caveats, as I may not have found them all if they have a common name or they have changed address, but I did my best. I tried to pick only individuals who have been on Twitter for some time and I deliberately overlooked people who were on BIG genome papers such as the first human genomes as this over-inflated the citation scores. I also captured whether the scientists were men or women. I had intended to collect more data but it took a long time and I therefore decided 40 would be enough to make a point. Please don’t take this as representative of my normal research rigor.

**Figure 1:**
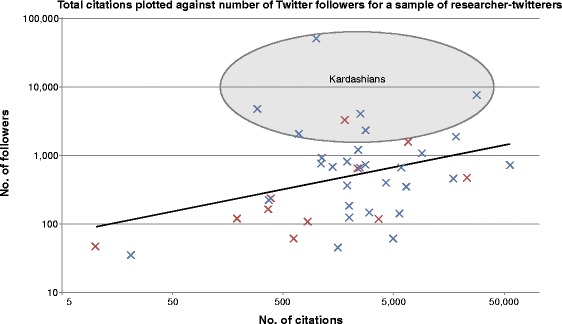
**Twitter followers versus number of scientific citations for a sort-of-random sample of researcher tweeters.** Red crosses represent female tweeters and blue crosses represent male tweeters. The black trendline describes the best fit to the data. Those individuals with a highly overinflated number of followers (when compared with the number predicted by the trendline) are highlighted by the area labeled Kardashians.

I took the number of Twitter followers as a measure of ‘celebrity’ while the number of citations was taken as a measure of ‘scientific value’ (we can argue about that another time). The data gathered are shown in Figure 1.

## Results

While aware that the analysis is flawed and lacks statistical rigor, it is a relief to see that there is some kind of positive trend in scientific value when compared with celebrity. The trend can be described by Equation 1:

$$ F\kern0.5em  = 43.3{C}^{0.32} $$

Where *F* is the number of twitter followers and *C* is the number of citations.

As a typical number of followers can now be calculated using this formula, I propose that the Kardashian Index (K-index) can be calculated as follows in Equation 2:

$$ K- index\kern0.5em =\kern0.5em \frac{F_{(a)}}{F_{(c)}} $$

Where *F*_(a)_ is the actual number of twitter followers of researcher X and *F*_(c)_ is the number researcher X should have given their citations. Hence a high K-index is a warning to the community that researcher X may have built their public profile on shaky foundations, while a very low K-index suggests that a scientist is being undervalued. Here, I propose that those people whose K-index is greater than 5 can be considered ‘Science Kardashians’; these individuals are highlighted in Figure 1.

## Discussion

In an age dominated by the cult of celebrity we, as scientists, need to protect ourselves from mindlessly lauding shallow popularity and take an informed and critical view of the value we place on the opinion of our peers. Social media makes it very easy for people to build a seemingly impressive persona by essentially ‘shouting louder’ than others. Having an opinion on something does not make one an expert. But on Twitter, for example, the ‘top tweet’ on any given subject will not necessarily come from an expert, it will come from the most followed person. If Kim Kardashian commented on the value of the ENCODE project, her tweet would get more retweets and favorites than the rest of the scientific community combined. Experts on the Syrian conflict will tell you how frustrating that can be.

I propose that all scientists calculate their own K-index on an annual basis and include it in their Twitter profile. Not only does this help others decide how much weight they should give to someone’s 140 character wisdom, it can also be an incentive - if your K-index gets above 5, then it’s time to get off Twitter and write those papers.

## Finally on a serious note

My introduction highlights the fact that women have a history of being ignored by the scientific community. Interestingly, in my analysis, very few women (only one in fact) had a highly inflated Twitter following, while most (11/14) had fewer followers than would be expected. Hence, most Kardashians are men! This ‘study’ does not prove that we, as a community, are continuing to ignore women, or if women are less likely to engage in self-promotion, but it is consistent with either or both of these scenarios.

If you would like to discuss this further please follow me on Twitter: @neilhall_uk. At the time of writing, my K-index in only marginally above 1. A few tweets linking me with the word ‘Kardashian’ should put my K-index through the roof.

## Abbreviation

K-index: Kardashian index
